# Do Students Really Use Internet Access for Learning in the Classroom?: Exploring Students’ Cyberslacking in an Indonesian University

**DOI:** 10.3390/bs9120123

**Published:** 2019-11-22

**Authors:** Ermida Simanjuntak, Nur Ainy Fardana Nawangsari, Rahkman Ardi

**Affiliations:** 1 Doctoral Program, Faculty of Psychology, Airlangga University, Surabaya 60286, Indonesia; nurainy.fardana@psikologi.unair.ac.id (N.A.F.N.); rahkman.ardi@psikologi.unair.ac.id (R.A.); 2 Faculty of Psychology, Widya Mandala Catholic University, Surabaya 60112, Indonesia

**Keywords:** cyberslacking, self-regulated learning, media multi-tasking efficacy, university students

## Abstract

University students, as ‘digital natives’, use the internet for learning in the classroom. However, the availability of internet access in the classroom becomes a challenge, because students also engage in non-academic internet access during lectures. The use of the internet during lectures for non-academic purpose is called cyberslacking. Self-regulated learning (SRL) and media multi-tasking efficacy (MME) are considered to be important factors contributing to cyberslacking. The participants in this study were students in a private university in Indonesia (N = 423). The results show that only self-regulated learning made any significant contribution to cyberslacking in the classrooms. Further research should be conducted to consider external factors, such as instructors’ contributions, classrooms circumstances and the university policy towards internet usage.

## 1. Introduction

The provision of internet access at a university is aimed to provide the opportunity for students to independently access online material resources with the aim of improving their mastery of the targeted learning materials [1]. The current landscape in university learning shows the presence of internet-related equipment: gadgets, laptops and smartphones, which students carry with them during class [2]. The methods of note-taking, using paper and pencil, employed by prior generations, have now changed to the use of laptops as tools for note-taking in the current learning environment [3]. The availability of internet access on campus provides challenges to lecturers in classrooms, owing to the tendency of students to browse non-academic content, such as social media, to update status or access games, and browsing other websites irrelevant to the learning materials being discussed [4,5]. Accessing the internet during class for such non-academic content, irrelevant to the learning objectives, is referred to as “cyberslacking” or “cyberloafing” [6,7].

Research shows that cyberslacking effects the results of student learning, and should be considered as an important issue for learning [8,9,10]. If students are busy accessing social media in the classroom, this will result in a decrease in their attention towards the learning materials being used [11]. A piece of research involving 269 students on several campuses in the USA, showed that 92% of them sometimes sent messages during class, with 30% of them doing this on a daily basis [2]. When students access non-academic content, they may have less comprehension of the learning materials, owing to a decrease in concentration, despite possibly having outstanding intellectual capacities [12]. 

Research on cyberslacking categorized several antecedents to this behaviour, in educational settings, such as ineffective lecturers, students, learning materials and learning environments [13]. Lecturers with effective teaching methods tend to make students more focused in the classroom which in turn discourages them from opening social media. [14]. From the perspective of learning materials, students who find that the materials are irrelevant to their needs, or hard to understand, tend to engage in cyberslacking during lectures [14]. From the perspective of the environment in class, students joining large classes tend to engage in inappropriate activities with their laptops [15]. The antecedents of cyberslacking behaviours, from the perspective of students, show that demographic factors influence students to engage in cyberslacking in class, such as learning motivation, locus of control, self-efficacy and self-regulation [13,16,17,18,19]. 

The power of self-regulation becomes the decisive factor in students engaging in cyberslacking, in spite of a less than supportive class environment [16,20]. Students who can regulate themselves well, in trying to achieve their learning objectives, will strive to focus on the learning materials to solve any distraction-arousing learning problems, including the inclination to engage in cyberslacking [20]. Another influencing factor is the conviction of the student to engage in multi-tasking will influence the tendency to use the internet in class, including the potential to engage in cyberslacking [21]. Research proves that the inclination to engage in media multi-tasking is not representative of the actual ability of the students to engage in media multi-tasking, although students having a conviction of their ability to engage in media multi-tasking will tend to commit to activities irrelevant to the lesson, using their laptops [16]. 

Referring to the roles of self-regulated learning and media multi-tasking elaborated upon above, this research aimed to ascertain the influences of self-regulated learning, and media multi-tasking efficacy factors, toward the cyberslacking behaviour of students. Research on cyberslacking in the university context in Indonesia, however, is still relatively rare, compared to research conducted on cyberslacking in the context of the field of employment [22,23]. Surveys on internet use in Indonesia show that 89% of Indonesian internet users are university students [24]. Additionally, surveys conducted by the Indonesian Internet Access Association (*Asosiasi penyelenggara Jasa Internet Indonesia -* APJII) found that 49.52% of the internet users in Indonesia are aged in the 19–34 years range, which covers the ages of most university undergraduate students [25]. Based on this, this research was aimed at disclosing the phenomenon of cyberslacking in the context of a university in Indonesia, by considering the factors of self-regulation and media multi-tasking efficacy. The research questions for this research were:

RQ1: Do self-regulated learning and media multi-tasking efficacy predict the cyberslacking behaviour of university students?

RQ2: What are the contributions of self-regulated learning, and media multi-tasking efficacy, toward the behaviour of cyberslacking behaviour of university students?

### 1.1. Literature Review on Academic Cyberslacking

Cyberslacking in educational settings is defined as the behavior of non-academic internet accessing, conducted by students during class time [5]. Forms of cyberslacking behaviours include sharing, shopping, real time updating, accessing online content, gaming and gambling [5]. The theory used in explaining cyberslacking is the social cognitive theory (henceforth, SCT) by Bandura [26]. SCT can explain the media use of a particular individual, determined by the aspects of behaviour (B), environment (E) and person (P). These three factors will influence one another in use of media, including the internet [26]. In the context of SCT, the influencing factor from the ‘person’ aspect (P) is self-efficacy and self-regulation [26]. Self-efficacy in online behavior is the conviction of the individual regarding his/her ability whilst accessing the internet [27,28]. Self-regulation related to online behavior is the ability of a person to regulate him/herself regarding the use of internet access.

### 1.2. Media Multi-Tasking Efficacy (MME) and Cyberslacking Behaviour in an Academic Context

Media multi-tasking efficacy (MME) is based on the SCT, regarding self efficacy, which refers to the conviction of an individual that he/she is able to use various media simultaneously [21]. A person with a conviction of being able to engage in multimedia multi-tasking will tend to engage with the media in his/her possession [29,30]. A student with the conviction of having the ability to engage in multi-tasking with laptop and cellular phone, will tend to access the internet using such devices in class [16,21]. Students, as “digital natives”, assume that their skills are high enough to enable them to access the internet while simultaneously listening to the lecturer’s explanations. This, however, does not always represent the actual truth, since they tend to overestimate their own capacity in this matter [31]. On the other hand, multi-tasking involving access to laptops and cellular phones impacts the ability of students to focus on the learning material at hand, owing to a failure of concentration encountered whilst simultaneously reading information on gadgets and attending to information offered in class [32]. However, not many pieces of research directly connect media multi-tasking with cyberslacking behaviour in class [23]. Thus, this research sought to observe the influence of media multi-tasking efficacy as a probable contributing factor to the behavior of cyberslacking in class. The proposed hypothesis is:

**(H1).**  *Media multi-tasking efficacy (MME) predicts cyberslacking behaviour by students in class.*

### 1.3. Self-Regulated Learning (SRL) and Cyberslacking in Higher Education

Self-regulation in the context of SCT is one of the important ‘person’ factors, owing to its influence on the purpose of that person [26]. In the context of internet access, self-regulation influences to what extent students have self-control regarding internet access in class [16]. The results of self-regulation, related to the ‘learning situation,’ is referred to as self-regulated learning (SRL): the ability of a person to direct his or her cognition, affection and behaviour to achieve his or her learning objectives [33]. Any person with learning objectives will try to direct him or herself in order to achieve those objectives, although the surrounding environment may be less than supportive of that objective [34]. The achievements of the learning objectives are related to the learning strategies applied, including planning, monitoring and regulation [33]. SRL includes factors such as motivation regulation, effort regulation, planning, attention focusing, task strategies, using additional resources and self-instruction [35].

Related to the behaviour of accessing the internet in class, students with high SRL will tend to be able to control themselves, resisting accessing the internet on subjects beyond the matters relevant to their lessons [36,37]. Someone with good SRL will tend to be motivated in monitoring his or her learning objectives, resulting in accessing the internet on matters relevant only to the achievement of the learning target. [38]. Thus, students with higher self-regulation may exhibit stronger control when accessing the internet for non-academic purposes, compared to students with low SRL. The direct connection between SRL and cyberslacking, however, has not yet become conclusive, since some studies show that there is a significant relationship between the two matters, whilst other studies showed only an insignificant relationship between them [20,23]. Thus, this research sought to examine the contribution of SRL factors toward cyberslacking behaviours. The second hypothesis for this research is:

**(H2).**  *Self-regulated learning predicts cyberslacking behaviours by students, in class.*

## 2. Materials and Methods

### 2.1. Participants

The participants for this research were university students of a private university in Surabaya, East Java, Indonesia (N = 423), there being 114 male participants and 309 female participants. The ages of the participants ranged from 17–26 years. The participants were students coming from the Faculties of Medicine, Nursing, Pharmacology and Psychology. 

### 2.2. Procedure

Data collection was conducted by distributing questionnaires to the participants. Questionnaires were distributed from 19 February to 8 March 2019. Some questionnaires were given after participants’ classes ended and some were distributed when participants took a break from class. Before the filling-in of the questionnaires, research assistants explained the purpose of the research to the prospective participants. If the participants agreed to participate in the survey, they then filled out the informed consent of participants in this study and continued to fill in the survey form. The questionnaire is anonymous in order to make the participants comfortable in completing it. The procedure of this study was approved by the Research Ethics Committee, Faculty of Psychology, Airlangga University. 

### 2.3. Instruments 

#### 2.3.1. Cyberslacking 

Cyberslacking was measured using a cyberslacking scale developed by Akbulut, Dursun and Donmez, and adapted into Indonesian [5]. This scale comprised 30 items and 5 cyberslacking indicators, including sharing, shopping, real time updating, accessing online content, and gaming/ gambling. Participants chose their answers from offered choices, ranging from “Never” to “to a Great Extent”. One of the sample items was “I check my friends’ social networking profiles”. Another sample on the shopping aspect was “I visit online shopping sites”. The Cronbach’s alpha coefficient on the cyberslacking scale was 0.925.

#### 2.3.2. Media Multi-Tasking Efficacy (MME)

Multi-media efficacy was measured using a media multi-tasking efficacy scale, adapted from the media multi-tasking self-efficacy scale developed by Jiun-Yu Wu, and adapted into Indonesian. The scale comprised 5 statement items and had a Cronbach’s alpha coefficient of 0.751. The scale consisted of 5 answer-alternatives, which were 1 (Not at all like me), 2 (Not much like me), 3 (Neutral), 4 (Somewhat like me) and 5 (Very much like me). A sample statement for this research was “I can surf the Internet for non-academic purposes while studying, and still study efficiently”.

#### 2.3.3. Self-Regulated Learning (SRL)

SRL was measured by using an SRL scale developed by Kadioglu, Uzuntiryaki and Aydin in 2011, and then adapted into Indonesian. The value of Cronbach’s alpha coefficient from the scale was 0.759 and it measured 7 indicators: motivation regulation, planning, effort regulation, attention focusing, task strategies, using additional resources and self-instruction. The SRL scale comprised 28 statement items, with answer options ranging from 1 to 5, i.e., Strongly agree, Agree, Slightly agree, Disagree and Strongly disagree. A sample of one statement item from this scale was “I underline important points while studying for a task”.

## 3. Results

The data was analysed using multiple regression analysis, to explore the contributions of media multi-tasking efficacy and self-regulated learning on the cyberslacking behaviour of the students. The descriptive data showed that the number of participants from the Faculty of Medicine was 148 (35%), the Faculty of Nursing was 94 (22.2%), the Faculty of Pharmacology was 74 (17.5%) and from the Faculty of Psychology was 107 (25.3%). The student participants comprised of freshmen, 77 students (18.2%); sophomores, 53 students (12.5%); juniors, 78 students (18.4%); seniors, 205 students (48.5%); plus 7 fifth-year students (1.7%), 2 sixth-year students (0.5%) and 1 seventh-year student (0.2%). 

Results also showed that most of the 384 student participants, (90.8%) admitted engaging in cyberslacking during class, and only 39 students (9.2%) did not admit this. On the question “How much time is spent on cyberslacking in class?” most participants, 140 students (33.1%), engaged in cyberslacking for 15 to30 minutes, 119 students (28.1%) did so for more than 15 minutes, 87 students (20.6%) did so for 30 to 60 minutes, and 77 students (18.2%) engaged in cyberslacking for more than 1 hour in class. The correlation data between cyberslacking academic, media multi-tasking and self-regulated learning can be seen in Table 1, below: 

The results in Table 2 show significant correlation between cyberslacking and self-regulated learning, while media multi-tasking efficacy did not correlate significantly with cyberslacking. Not all aspects of self-regulated learning are significant to cyberslacking. Significant correlations were only found in the aspects of task strategies and self-instruction in self-regulated learning. There are no significant correlations between motivation regulation, planning, effort regulation, attention focusing and using additional resources. 

To test the research hypothesis, multiple regression analysis was conducted, considering the aspects of gender, age, and batch, shown in Table 2. 

Model 1 includes demographic factors such as gender, age and year. Results show that demographic factors (gender, age and batch) do not correlate to cyberslacking behaviour. There are no significant differences of cyberslacking behaviour between males and females, or for age and between batches. Model 2 explains that there are no significant correlations between demographic factor and media multi-tasking self-efficacy with cyberslacking behaviour. It means that students who were confident with their ability to do media multi-tasking also do cyberslacking during lectures as much as students who are less confident in media multi-tasking.

A significant model to explain cyberslacking by students was found only in Model 3 (see Table 2, above), when the variable of self-regulated learning was included in it, by considering the demographic factors of gender, age and batch. Model 3 explained 18% of the cyberslacking variation engaged in by students in class. In this research, the demographic variables of age, gender, and batch were not correlated to cyberslacking. Results showed that the first hypothesis was not significant, meaning that media multi-tasking efficacy does not predict the cyberslacking behaviour of students in class. However, results also showed that the second hypothesis was accepted, i.e., that self-regulated learning does predicts cyberslacking by students, during lectures. 

## 4. Discussion

### 4.1. Do Self-Regulated Learning and Media Multi-Tasking Efficacy Influence the Cyberslacking Behaviour of Students?

Results showed that self-regulated learning significantly does predict “cyberslacking” by students. This is in line with the notion of SCT, which states that self-regulation is an important factor in online behaviour [26]. It is interesting to notice that there is a positive correlation between SRL and cyberslacking, indicating that the higher the SRL students possess, the higher their tendency to engage in cyberslacking. This is in opposition to the findings in previous studies, stating that self-regulation may decrease problematic internet access behaviour [16,26]. From the correlation aspects of SRL and cyberslacking, there was a significant relationship between task strategies and cyberslacking. The aspect of task strategies was included with the ability of students to formulate strategies in completing the tasks at hand [35]. Some students mentioned that they will do their assignments that they consider important in other lectures, rather than listening to lectures that they are attending. Regarding students’ academic life that have many assignments, students must have a strategy to finish all their assignments and sitting in the class while doing other lectures’ assignments can be considered as task strategy [35]. During finishing the other lectures’ assignments, students will chat and discuss with their friends using social media and this is included in cyberslacking behaviour. This is in line with the fact that the internet, especially social media, is a tool to find help and support when encountering difficult situations [14]. Cyberslacking may be perceived as a means in finding help to understand difficult learning materials [14]. Results further proved that young people used social media as a means to obtain support from their peers, and in dealing with difficulties, including learning or adaptation difficulties [39]. 

Another possible explanation regarding positive correlation between self-regulated learning and cyberslacking is that cyberslacking might be perceived as one of the ways to quickly escape the feeling of boredom, in order, later on, to refocus on the learning materials being discussed by the lecturer [14]. Some students mentioned that some lecturers can not explain the learning material well and their method of teaching is boring. In that case, engaging in cyberslacking for some minutes is a fast solution to overcome boredom in the classroom so that they can refocus again on the learning materials. The lecturers have to create learning situations that can attract students’ attention to the learning material [40,41]. Some lecturers can not conduct such attractive learning situations and students tend to be bored in the classrooms. Cyberslacking for some students can be regarded as one of the possible solutions to overcome boredom as part of self-regulated learning on the part of task strategies and self-instruction [35].

As regards to the first hypothesis, concerning media multi-tasking efficacy (MME) and cyberslacking, results showed that there was no significant effect from MME on cyberslacking. This result was in opposition to previous studies, which found that MME was affecting students’ online behaviour [21,29,31]. This research, conversely, found that students with both high and low conviction rates regarding the ability to perform media multi-tasking did engage in “cyberslacking” in class. Related to the descriptive results, 90.8% of the total number of participants engaged in cyberslacking in class, indicating that conviction of having the ability to engage in media multi-tasking was not a significant factor in the triggering of cyberslacking behaviour.

### 4.2. What Were the Contributions of Self-Regulated Learning, and Media Multi-Tasking Efficacy towards Cyberslacking Behaviour by Students?

Results showed that only the self-regulated learning variable contributed to cyberslacking by students, whilst media multi-tasking did not significantly contribute to such behaviour. This may well be worth consideration in understanding the application of SCT to explain cyberslacking, in that self-regulation of online behaviour in learning has a stronger effect than self-conviction regarding multi-tasking [16,26]. Self-regulated learning only contribute 18% to cyberslacking behaviour and it can be regarded as part of doing task strategy [35]. Students will do cyberslacking in order to communicate with their friends to finish other lecturers’ assignments and overcome boredom so that they can refocus again to the lectures that they attend. Those two facts from results interview can be regarded as task strategy in self-regulated learning [35].

There were several limitations on this research. Firstly, the tool used to collect data on media multi-tasking efficacy was the use of the social media Facebook. This may have affected the results, since most university students in Indonesia prefer to use Instagram, Line and WhatsApp. It is therefore necessary to develop a measurement tool for media multi-tasking efficacy which is more appropriate to the behavioural characteristics of Indonesian students. Second, the role of the lectures were not fully explored in this study. Some participants mentioned in the open questions that they were bored during lectures so that they do cyberslacking. However, this study did not include external factors such as the role of lecturers in classrooms or extrinsic motivation triggered by lecturers. Another limitation to be considered was the fields of science of the participants, which were limited to those from the Faculties of Medicine, Nursing, Pharmacology and Psychology. The research on the use of technology showed the interconnection between the different fields of science, i.e., the ‘hard’ and ‘soft’ sciences, and/or the ‘pure’ and ‘applied’ sciences, regarding online behaviour [4,17]. The variety in the fields of science could be a consideration for the next researchers studying cyberslacking by students. Related to SCT, the triggering factors from the environment for cyberslacking (E), such as the role of lecturers, learning methods, class situations, peers, and other environmental factors, need to be considered for further studies.

## 5. Conclusions

Results of this research showed that self-regulated learning does predict the “cyberslacking” behaviour of students, whilst media multi-tasking efficacy does not contribute to the students’ cyberslacking such behaviour. The SRL aspect related to cyberslacking is task strategy, meaning that cyberslacking may act as one of the strategies used by students to complete learning assignments. Further research should consider other external environmental factors, such as classroom ambience, attitudes of instructors, and policies regarding internet usage. 

## Figures and Tables

**Table 1:** Correlation between cyberslacking, media multi-tasking efficacy and self-regulated learning (N = 423).

Variables	M	SD	Var 1	Var 2	Var 3
Var 1 Cyberslacking	77.4	16.45			
Var 2 Media multi-tasking efficacy	17.2	5.14	0.087		
Var 3 Self-regulated learning	96.22	10.25	0.124 *	−0.026	-
Motivational regulation	13.48	13.48	−0.026	0.087	-
Planning	14.09	14.09	0.032	0.024	-
Effort regulation	14.87	14.87	0.013	0.085	-
Attention focussing	14.15	14.15	0.071	−0.018	-
Task strategies	13.35	13.35	0.139 *	−0.010	-
Using additional resources	12.58	12.58	0.040	−0.082	-
Self-instruction	13.71	13.71	0.152 *	−0.031	-

* *p* < 0.01.

**Table 2:** Results of multiple regression analysis (N = 423).

Models	*B*	Standard Error *b*	β	R^2^	R^2^ Change
**Model 1**				0.007	0.000
Gender	0.468	1.808	0.013		
Age	0.020	0.764	0.002		
Year	1.135	0.957	0.084		
**Model 2**				0.014	0.005
Gender	0.402	1.804	0.011		
Age	0.091	0.764	0.008		
Year	1.041	0.956	0.077		
Media multi-tasking efficacy	0.271	0.156	0.085		
**Model 3**				0.180 *	0.032 *
Gender	0.055	1.794	0.001		
Age	−0.056	0.759	−0.005		
Year	1.331	0.954	0.099		
Media multi-tasking efficacy	0.280	0.155	0.088		
Self-regulated learning	0.216	0.078	0.134 *		

* *p* < 0.01.
